# Diversity patterns of selected Andean plant groups correspond to topography and habitat dynamics, not orogeny

**DOI:** 10.3389/fgene.2014.00351

**Published:** 2014-10-10

**Authors:** Jens Mutke, Rana Jacobs, Katharina Meyers, Tilo Henning, Maximilian Weigend

**Affiliations:** ^1^Nees Institute for Biodiversity of Plants, Rheinische Friedrich-Wilhelms-Universität BonnBonn, Germany; ^2^Botanischer Garten und Botanisches Museum Berlin-Dahlem, Freie Universität BerlinBerlin, Germany

**Keywords:** tropical Andes, range size, latitudinal gradient, altitudinal gradient, endemism, plant diversity

## Abstract

The tropical Andes are a hotspot of biodiversity, but detailed altitudinal and latitudinal distribution patterns of species are poorly understood. We compare the distribution and diversity patterns of four Andean plant groups on the basis of georeferenced specimen data: the genus *Nasa* (Loasaceae), the two South American sections of *Ribes* (sect. *Parilla* and sect. *Andina*, Grossulariaceae), and the American clade of *Urtica* (Urticaceae). In the tropical Andes, these often grow together, especially in (naturally or anthropogenically) disturbed or secondary vegetation at middle to upper elevations. The climatic niches of the tropical groups studied here are relatively similar in temperature and temperature seasonality, but do differ in moisture seasonality. The Amotape–Huancabamba Zone (AHZ) between 3 and 8° S shows a clear diversity peak of overall species richness as well as for narrowly endemic species across the groups studied. For *Nasa*, we also show a particular diversity of growth forms in the AHZ. This can be interpreted as proxy for a high diversity of ecological niches based on high spatial habitat heterogeneity in this zone. Latitudinal ranges are generally larger toward the margins of overall range of the group. Species number and number of endemic species of our taxa peak at elevations of 2,500–3,500 m in the tropical Andes. Altitudinal diversity patterns correspond well with the altitudinal distribution of slope inclination. We hypothesize that the likelihood and frequency of landslides at steeper slopes translate into temporal habitat heterogeneity. The frequency of landslides may be causally connected to diversification especially for the numerous early colonizing taxa, such as *Urtica* and annual species of *Nasa*. In contrast to earlier hypotheses, uplift history is not reflected in the pattern here retrieved, since the AHZ is the area of the most recent Andean uplift. Similarly, a barrier effect of the low-lying Huancabamba depression is not retrieved in our data.

## INTRODUCTION

### ANDEAN DIVERSITY GRADIENTS

The northern and central Andes have been identified as globally outstanding centers of plant diversity across life forms and phylogenetic groups (Gentry, 1982; Myers et al., 2000; Barthlott et al., 2005; Mutke et al., 2011). Andean biodiversity has primarily latitudinal and altitudinal dimensions. There have been several studies addressing altitudinal patterns of diversity in the Andes. For birds, a widely cited study by Terborgh (1977) shows a monotonic decrease of bird species richness with elevation in the Peruvian Andes. In the Peruvian flora, epiphytic orchids show highest species richness at lower and mid elevations up to 2,000 m above sea level (m a.s.l.). Terrestrial orchid diversity peaks around 2,000–2,500 m a.s.l. and overall species richness of orchids peaks at ca. 1,500–2,000 m a.s.l. (Ibisch et al., 1996). Similar patterns were documented by Küper et al. (2004) for Ecuadorian epiphytic plants. In contrast, overall diversity of tree taxa and of the entire flora across taxonomic groups and life forms peaks at low elevations (<500 m a.s.l.) both in Peru and Ecuador (Braun et al., 2002; Jørgensen et al., 2011; Mutke, 2011) – showing parallel trends to the available surface area per elevational zone. Plant endemism in Peru peaks at 1,500–3,000 m across life forms, a figure based on the overall predominance of epiphytes, terrestrial herbs, and shrubs amongst endemic taxa (Van Der Werff and Consiglio, 2004). “Density” of endemic species (species per 1,000 km^2^) in their study was even found to be more than 10 times higher at 2,000–3,500 m than in the Amazonian lowlands (0–500 m, Van Der Werff and Consiglio, 2004) – however, this was computed based on a linear species-area relationship, which is at least problematic. Diversity of predominantly tropical, mostly shrubby families (Acanthaceae, Araceae, Melastomataceae, and Palmae) in Bolivia peaks at relatively lower elevations (Kessler, 2001, 2002). Similar results have been found by other authors and overreaching patterns for the Andes, based on the most up-to-date taxonomic information available, support this conclusion (Jørgensen et al., 2011). Topographical and habitat heterogeneity together with the water and energy balance are considered as the overall most prominent factors influencing levels of plant diversity at the geographical scale (Mutke et al., 2001; Braun et al., 2002; Kreft and Jetz, 2007; Jørgensen et al., 2011), but their detailed interplay in the Andes has so far eluded analysis.

### EVOLUTION OF TROPICAL ANDEAN TAXA

Several recent studies try to identify the evolutionary processes that are at the basis for the diversity gradients observed. Gentry (1982) already postulated an “explosive speciation and adaptive radiation” in shrubs and epiphytes as a direct consequence of Andean uplift, especially in the northern Andes. It has been hypothesized that Andean uplift was the causal agent for increased diversification in the shrub-genus *Hedyosmum*, a genus of some 44 species in the Chloranthaceae (Antonelli and Sanmartín, 2011). There, it is assumed that uplift itself separated populations and provided the basis for allopatric speciation, since diversification in *Hedyosmum* apparently took place in parallel to Andean uplift. Similarly, diversification patterns in Rubiaceae are hypothesized to correlate closely to paleogeography in the Andes and the Amazon, with a long-lasting separation of the northern and the central Andes (Antonelli et al., 2009). Similar arguments have been brought forward for Andean *Macrocarpaea* (Struwe et al., 2009). Conversely, several publications show that the diversification of particular high Andean clades predates the formation of the current habitats (Bell and Donoghue, 2005; Hershkovitz et al., 2006; Palazzesi et al., 2009, 2012; Emadzade et al., 2010). Another, by now widely documented, phenomenon is the very recent and explosive radiation of high Andean (Páramo and Puna) groups. It appears to have essentially taken place after the main Andean uplift (e.g., *Gentianella* and *Halenia, Hypericum, Lupinus, Valeriana*: von Hagen and Kadereit, 2001; Kadereit and von Hagen, 2003; Bell and Donoghue, 2005; Hughes and Eastwood, 2006; Nürk et al., 2013). Overall, a wide range of high Andean (Páramo and Puna) taxa seem to have radiated extensively and very recently (Madriñán et al., 2013). These examples might indicate that mid-elevation diversity predates high-elevation diversity and has a strong historical component. However, upper slope taxa, from elevations of 2,000–3,500 m, have not been extensively studied so far and the phylogenetic studies do not include an analysis of the detailed altitudinal and latitudinal diversity patterns of the plant groups concerned. In this altitudinal band we would expect to find a strong historical signal in latitudinal diversity, especially in high and mid-elevation plant groups, since Andean uplift took place in dramatically different time periods, with the central Andes as the oldest part of the tropical Andes, followed by an uplift of the northern Andes and – last of all – an uplift of the connection of these two in the Amotape–Huancabamba Zone (AHZ; Hoorn et al., 2010). It is therefore surprising that two typical mid-slope groups of shrubby angiosperms, namely *Macrocarpaea* (Struwe et al., 2009) and Iochrominae (Solanaceae: Smith and Baum, 2006), appear to have centers of diversity and possibly ancestral areas in this AHZ, which is at odds with geological history.

### THE AMOTAPE–HUANCABAMBA ZONE – PHYTOGEOGRAPHICAL BARRIER OR DISTINCT PHYTOGEOGRAPHICAL REGION?

This region along the border of Ecuador and Peru is of particular interest, because of many Andean plant groups with elevated levels of diversity and narrow endemicity. It has been variously termed northern Peruvian Low, Huancabamba Deflection, Piura Divide, and the Huancabamba Depression, and is nowadays generally termed AHZ (Young and Reynel, 1997). This region, and most importantly the lowest part of the Andes in this region (lowest pass at 5.5° S) has frequently been referred to as a barrier for the dispersal of Andean plants (Vuilleumier, 1968; Molau, 1988; Prance, 1989), most recently by Richter et al. (2009). Berry (1982) was probably the first to argue against the theory of a phytogeographical barrier and for the recognition of a distinct phytogeographical region in this part of the Andes. Evidence to support this latter argument has been brought forward by a series of publications based on distribution data of a small set of Andean plant groups (Weigend, 2002, 2004a; Weigend et al., 2005; Struwe et al., 2009). Some studies presenting biogeographical conclusions in support of a biogeographic barrier give no explicit source of distribution data at all (Bonaccorso, 2009; Chaves et al., 2011) and can be safely disregarded. Also, most studies on latitudinal diversity patterns in individual plant groups usually take pre-defined geographical units as the basis for a distributional analysis, i.e., either dividing the tropical Andes into two units (north and south of the Huancabamba deflection: Molau, 1988; Cosacov et al., 2009; Antonelli and Sanmartín, 2011) or three regions (northern Andes, AHZ, and central Andes: Weigend, 2002, 2004a; Weigend et al., 2005; Smith and Baum, 2006; Struwe et al., 2009). Even more narrowly defined geographical units are followed in some botanical (Antonelli et al., 2009) and zoological (Weir, 2009) studies. In all these cases, the coding influences the patterns found, since fine-scale recognition of distribution limits is impossible when taxa are *a priori* assigned to geographical units. All taxa only found (somewhere) in one of the pre-defined units seem to underscore the presence of a biogeographical barrier between the units, and this is the grossly erroneous interpretation that has generally been provided.

### DATA FOR THE PRESENT ARTICLE

There is often a strong collection bias in published distribution data and the main strong point of the present study is that the distribution data from herbarium material have been extensively supplemented by field studies between 1993 and 2012, covering especially the most poorly known parts of Peru (eastern slope, northern Peru), so that the underlying data are rather extensive. Also, all plant determinations in our study are based on critical taxonomical work (comparison of types, revisionary work), so that underreporting is a relatively small factor and misidentifications are minimized. The study groups appear to be particularly suited to a study of the diversification patterns in the Andes, since they are present throughout the tropical Andes, two reach into Patagonia in the South (*Ribes, Urtica*). They are also most common at intermediate elevations. As mentioned above, these are of particular interest as far as diversification patterns are concerned and at the same time are the least understood region. All three groups are most diverse in disturbed, open habitats and secondary forest, relatively rare in primary forests and thus represent an ecological group largely neglected in previous biogeographical studies. At elevations between 2,000 and 4,000 m a.s.l. in the tropical Andes it is common to find representatives of *Ribes* subg. *Parilla* sect. *Andina*, *Nasa,* and *Urtica* growing together, since they share overall similar requirements. To a lesser extent, this is also true for *Urtica* and *Ribes* subg. *Parilla* sect. *Parilla* in the southern Andes.

### THE GENUS *Nasa*

Monophyletic *Nasa* belongs to the predominantly Neotropical plant family Loasaceae (Weigend, 2003). Currently, 128 taxa (98 species) are recognized, with a clear center of diversity in northern Peru (Weigend, 2003), where micro-allopatric subspecies and species are common (Dostert and Weigend, 1999; Henning and Weigend, 2009a). *Nasa* phylogeny is not fully resolved, but the group can be roughly subdivided into a range of informal species groups, largely corresponding to growth habit and ecological preferences. Some of these have been confirmed by molecular data (Weigend et al., 2004; Weigend and Gottschling, 2006). *Nasa* shows a range of different growth forms, which more or less closely correspond to ecological preferences: annuals, biennials, and subperennials are from disturbed and/or highly seasonal habitats, deciduous shrubs are found on steep scree slopes, whereas the evergreen shrubs are mostly typical of the upper limits of the cloud forest (subpáramo habitats), rhizomatous perennials are largely restricted to high-Andean grasslands, whereas stoloniferous and lianescent taxa are found in secondary forest. Growth habit is thus a good proxy for the vegetation type and dynamics that the species are found in (for details see Weigend and Rodríguez, 2003; Weigend et al., 2003; Weigend, 2004b; Henning and Weigend, 2009a; Henning et al., 2011).

### THE GENUS *Ribes* SUBGENUS *Parilla*

*Ribes* is a largely north-temperate genus in monogeneric Grossulariaceae and is present in South America with two different clades, both of which are exclusively berry-fruited (bird-dispersed), dioecious shrubs, classified in subgenus *Parilla*. *Ribes* subg. *Parilla* sect. *Andina* ( = *Ribes* sect. *Andina*) is largely restricted to the tropical Andes while *Ribes* subg. *Parilla* sect. *Parilla* ( = *Ribes* sect. *Parilla*) is restricted to the southern Andes (for details: Janczewski, 1907; Weigend et al., 2002; Weigend, 2007). The two sections are not closely related to each other and represent independent introductions from North America. *Ribes* sect. *Andina* contains 43 species overall (four of them undescribed) and extends from northern Argentina into Central America with a single species (Weigend and Binder, 2001a). The bulk of the species occur in the tropical Andes, where narrowly endemic species are common (Freire Fierro, 2004; Weigend et al., 2010). The shrubs are important components at the upper margins of the cloud forest and in the subpáramo formation, with many species found in secondary forests and only a few of them in the undergrowth of primary forests. They are also found in isolated patches at the base of rocks in Páramo and Puna vegetation. *Ribes* sect. *Parilla* comprises nine species overall (Weigend et al., 2008), and is restricted to Chile and Argentina, where it is common both in the forest undergrowth and at the upper limit of Patagonian forests.

### THE GENUS *Urtica*

The genus *Urtica* (Urticaceae) is predominantly north-temperate in distribution, but is represented with a total of 21 species in South America (Weddell, 1856, 1869; Weigend and Luebert, 2009). The members of *Urtica* are predominantly perennial, sometimes stoloniferous/rhizomatous, monoecious herbs from disturbed sites and secondary forest. Their one-seeded fruits are efficiently dispersed by animals and wind. South American taxa fall into two unrelated clades: *Urtica gracilis* s.l. with four subspecies in the American Cordillera between the northern USA and Chile and with one subspecies in temperate North America (Henning et al., 2014). *U. gracilis* s.l. is here excluded from the analysis, as representing a minor, highly disjunct component. The group here considered is monophyletic “American *Urtica*” (Farag et al., 2013; Henning et al., 2014) with a total of 20 species ranging from the eastern USA down to Argentina and Chile, but with the bulk (14 species) restricted to South America. It is sister to a Macaronesian-Mediterranean clade (Farag et al., 2013; Henning et al., 2014).

### THE AIM OF THE PRESENT STUDY

The aim of this study is an improved understanding of the patterns of diversity and endemism in these typical, diverse Andean plant taxa, based on actual latitudinal and altitudinal record data rather than assigning them to “units.” The study addresses the following questions:

1. Which latitudinal and altitudinal patterns of diversity and endemism can be found in these mid-elevational taxa?
2. Is there correspondence between current patterns of distribution and diversity with climate, uplift history, and topography?
3. Is the AHZ a barrier for north–south dispersal of mid-elevation taxa?

## MATERIALS AND METHODS

### SPECIES DATA

The distribution data was collected during the last two decades working on several taxonomic papers. For the genus *Nasa*, our dataset, which was established during preparatory work for a revision in the Flora Neotropica, includes 1,151 records for all 128 taxa (98 species). Published data are taken from Weigend (1996, 2000a,b, 2001, 2004a,b, 2012b), Weigend et al. (1998, 2003), Dostert and Weigend (1999), Rodríguez and Weigend (1999, 2004, 2006), Weigend and Rodríguez (2001, 2002, 2003), Rodríguez et al. (2002), Henning and Weigend (2009a,b, 2011) and Henning et al. (2011), supplemented with some additional herbarium records. The data for *Ribes* are based on revised herbarium specimens and include 1,189 samples for the 56 species of *Ribes* subg. *Parilla*. Published distribution data are found in Jørgensen and León-Yánez (1999), Weigend and Binder (2001a,b,c), Freire Fierro (2004), Weigend et al. (2005, 2010); Weigend and Rodríguez (2006), and Weigend (2008, 2012a). For the 20 species of “American *Urtica,*” 592 samples are included in the dataset, based on Navas (1961), Soraru (1972), Weigend et al. (2005), and Weigend and Luebert (2009), unpublished specimen records.

Especially many of the older specimens do not provide exact geographic coordinates. Thus, we had to georeference these based on the locality information stated on the herbarium labels with the help of spatial databases such as geonames.org, google earth, and google maps. We were able to assign coordinates with sufficient spatial resolution to 1,038 out of the 1,151 specimens for *Nasa*, 994 out of 1,189 samples for *Ribes*, and 497 out of the 592 specimens for *Urtica*. For more than 90% of the specimens of *Nasa* and *Urtica* we were able to assign coordinates with an estimated error of less than ±5 km. For *Ribes* specimens, this is true for only ca. 60% of the specimens. However, the accuracy was high enough for all records for the mapping in 50 km × 50 km grid cells and 1° latitudinal bands (see below). For the information on the altitude, where the specimen was sampled, we always used the original information stated on the herbarium label.

No phylogenies with species level resolution are available for the groups studied in this paper. The monophyly of *Nasa* was demonstrated by Weigend et al. (2004) and Weigend and Gottschling (2006). The phylogeny of the genus *Ribes* by Weigend et al. (2002) supports the independence and monophyly of Chilean and Argentinian sect. *Parilla* from the all tropical Andean species in the sect. *Andina*. The monophyly of “American *Urtica*” was demonstrated by Farag et al. (2013) and Henning et al. (2014).

**FIGURE 1:**
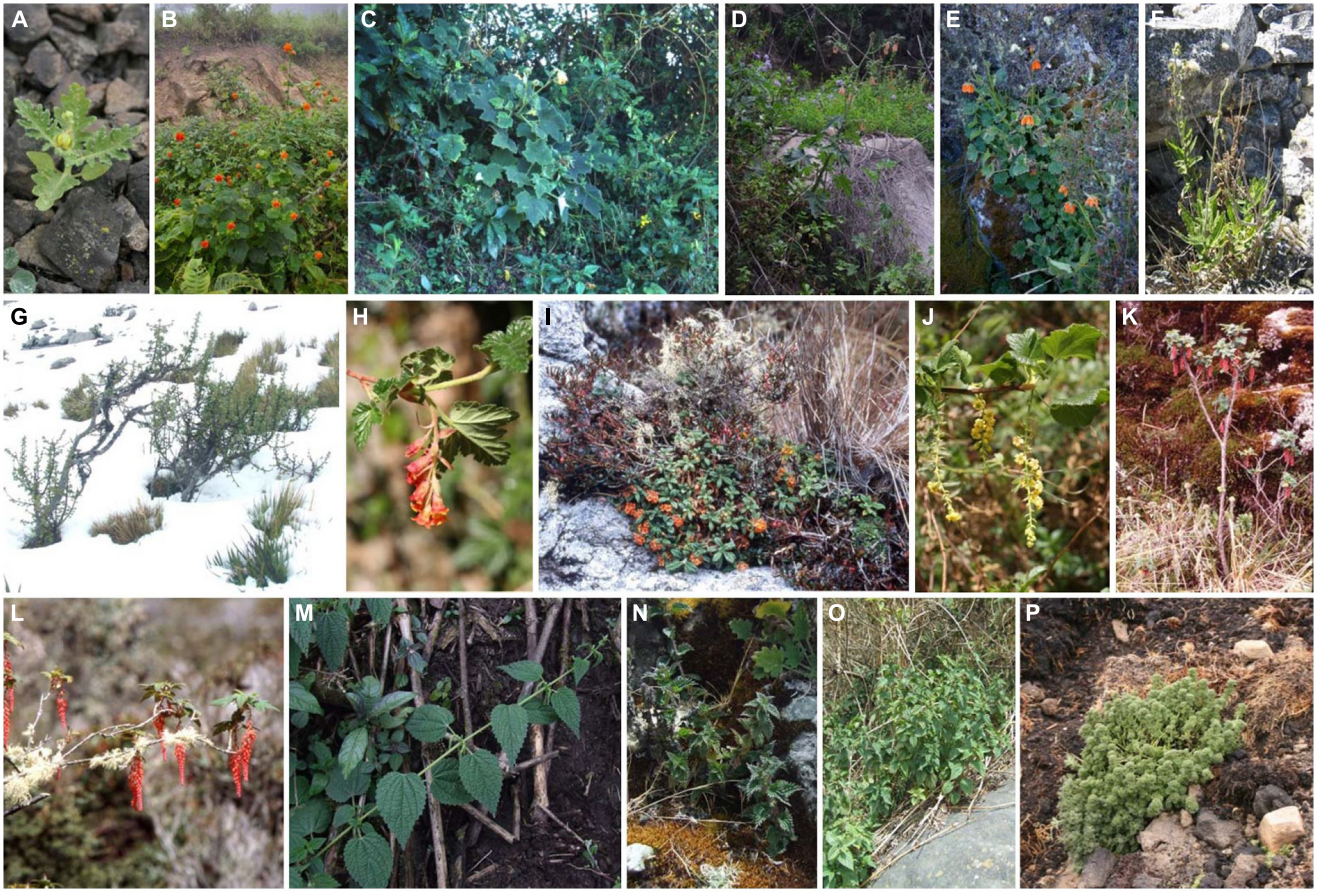
**Diversity of life forms of the Andean Genera *Nasa* (Loasaceae, **A–F**), *Ribes* subgenus *Parilla* (Grossulariaceae, **G–L**), and *Urtica* (Urticaceae, **M–P**). (A)** *Nasa urens* (annual), **(B)** *N. olmosiana* (annual), **(C)** *N. weberbaueri* (evergreen shrub), **(D)** *N. magnifica* (biennial), **(E)** *N. ranunculifolia* subsp. *macrorrhiza* (rhizomatous perennial herb), **(F)** *N. sanagoranensis* (deciduous shrub), **(G)** *Ribes cuneifolium*, **(H)** *R. weberbaueri*, **(I)** *R. frankei*, **(J)** *R. viscosum*, **(K)** *R. hirtum*, **(L)** *R. macrobotrys*, **(M)** *Urtica longispica*, **(N)** *U. echinata*, **(O)** *U. leptophylla*, **(P)** *U. flabellata*.

Coding of life history/growth form based on field observations (compare **Figure 1**) and cultivation data is straight forward for *Urtica* (annual herb, perennial herb, lianescent shrub) and *Ribes* (cushion plant, shrub, lianescent shrub), but more problematical in *Nasa*. *Nasa* shows a wide range of growth forms, only some of which are readily defined: biennial herbs, and perennial respectively deciduous shrubs, lianescent shrubs, stoloniferous versus rhizomatous perennial herbs (Weigend, 2001; Henning et al., 2011; compare **Figures 1C–F**). The categories annual and subperennial are more difficult to distinguish: “ephemeral” annuals, such as *Nasa urens* (**Figure 1A**) and *N. chenopodiifolia* are very short-lived plants flowering for only a few weeks and then passing into fruit. Larger annual species, such as *N. olmosiana* (**Figure 1B**), may flower for several months before finally dying after producing fruits for several consecutive months. Other taxa, such as *N. dilloniana*, live for anything up to a year and—under ideal conditions—decidedly longer. Unlike the taxa here defined as shrubs or perennial herbs, they lack any vegetative renewal growth, i.e., all lateral shoots immediately pass into flower, so that they are continuously in flower from reaching maturity and do not tolerate any interruption of the growing season. This group of plants is therefore here classified as “subperennial” —they ultimately succumb to their own weight or old age before reaching an age of ca. 2 years and lack any ability for vegetative regeneration.

### GIS DATA

To analyze diversity patterns of the three genera at different spatial scales we used equal-area grids (Behrmann projection) with 200 km × 200 km and 50 km × 50 km resolution (40,000 and 2,500 km^2^ grid cells). Climate data was taken from the WORLDCLIM data set (Hijmans et al., 2005), a global data set with a spatial resolution of 30”, which equals ca. 1 km^2^ at the equator. To characterize species distributions, we used elevation data from the GTOPO30 dataset, which has 30” spatial resolution, as well (U.S. Geological Survey, 1996). For the analyses of differences in slope along latitudinal and altitudinal gradients in the tropical Andes, we used the higher resolution SRTM Elevation data with 90 m resolution (Jarvis et al., 2008), employing the Surface Analysis toolbox of the Spatial Analyst tools in ArcView. Mean slope values and mean elevations are queried per 10 km × 10 km grid cells using the Zonal Statistics tools in ArcView.

### ANALYSES

Data analyses and production of the graphics were performed in R Version 3.0.0 (R Development Core Team, 2013). The threshold separating widespread and restricted range species were here set at 2° latitudinal range (ca. 220 km). Unfortunately, small scale spatial heterogeneity in the steep terrain of the Andes results in quality issues of the available climate datasets. Together with potential spatial errors of the georeferencing process this precluded more in-depth statistical analysis and modeling of diversity patterns for our study with regard to fine-scale climate parameters. For the visualization of the distribution and diversity patterns on maps and for the overlay with environmental data, we used ArcView 9.3 (Environmental Systems Research Institute [ESRI], 2008).

## RESULTS

### LATITUDINAL AND ALTITUDINAL PATTERNS OF DIVERSITY

**FIGURE 2:**
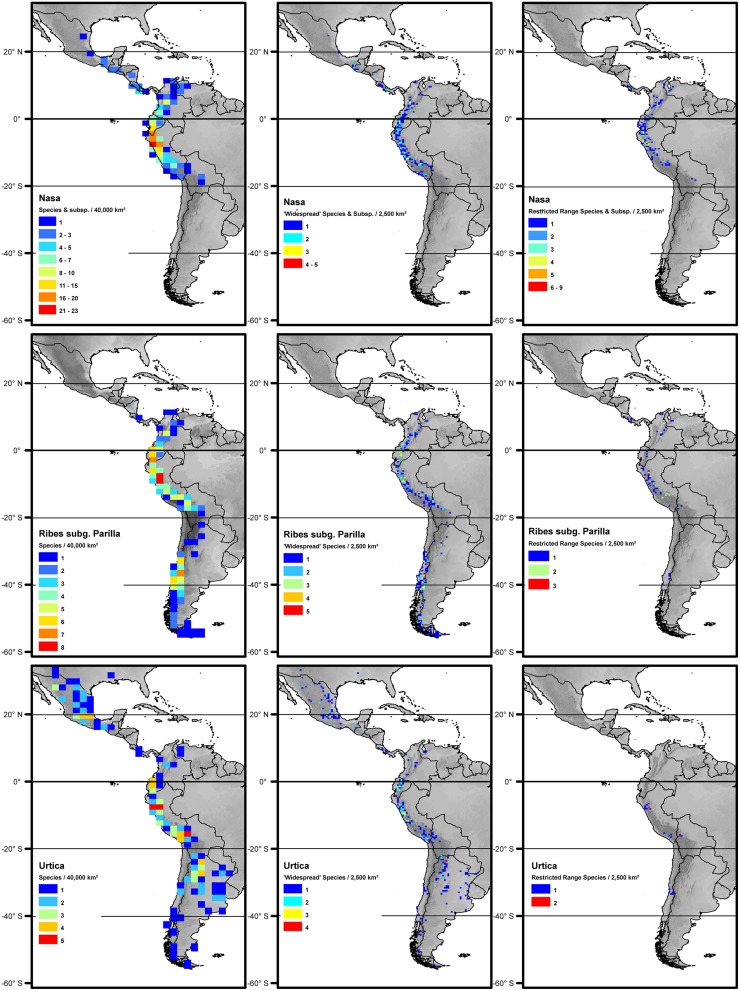
**Overall species richness and richness of restricted range species (less than 2° latitudinal range) and more widespread species (more than 2° latitudinal range) of *Nasa* (top), *Ribes* (center), and *Urtica* (bottom).** The diversity patterns are mapped in a 200 km** ×** 200 km grid (40,000 km^2^ grid cell size, maps on the left) respectively a 50 km** ×** 50 km grid (2,500 km^2^ grid cell size) using Behrmann projection. Topography is based on the GTOPO30 dataset (U.S. Geological Survey, 1996).

**FIGURE 3:**
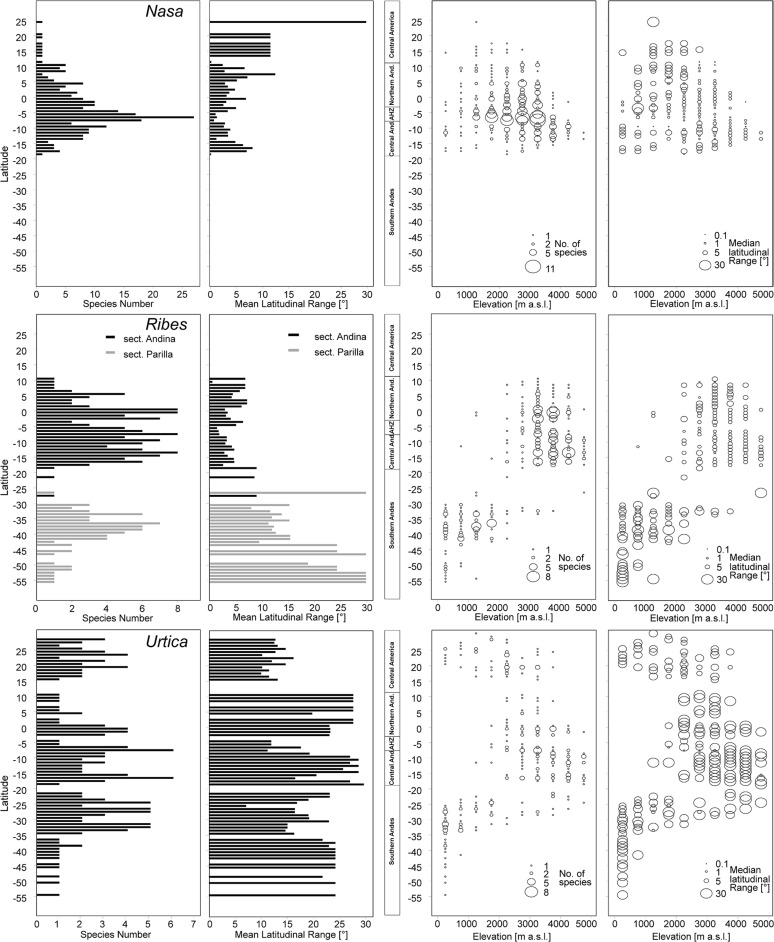
**Latitudinal and altitudinal gradients of species richness and range size for *Nasa* (top), *Ribes* subg. *Parilla* (center), and the “American *Urtica*” **(bottom)** in Latin America**.

All three tropical groups studied here have a center of diversity in northern Peru in the area called AHZ (ca. 3–8° S; **Figures 2** and **3**). This area is as well a center for restricted range species of all three genera. Looking at the detailed altitudinal and latitudinal diversity patterns in **Figure 3**, the highest diversity of *Ribes* sect. *Andina* can be found slightly displaced southward and to higher elevations.

**FIGURE 4:**
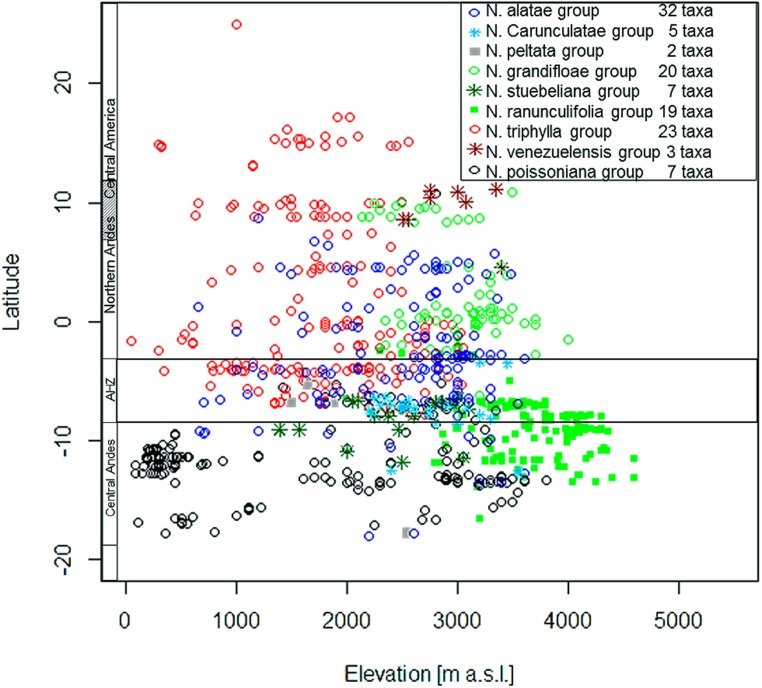
**Latitudinal and altitudinal distribution of the 11 infrageneric groups of the genus *Nasa***.

**FIGURE 5:**
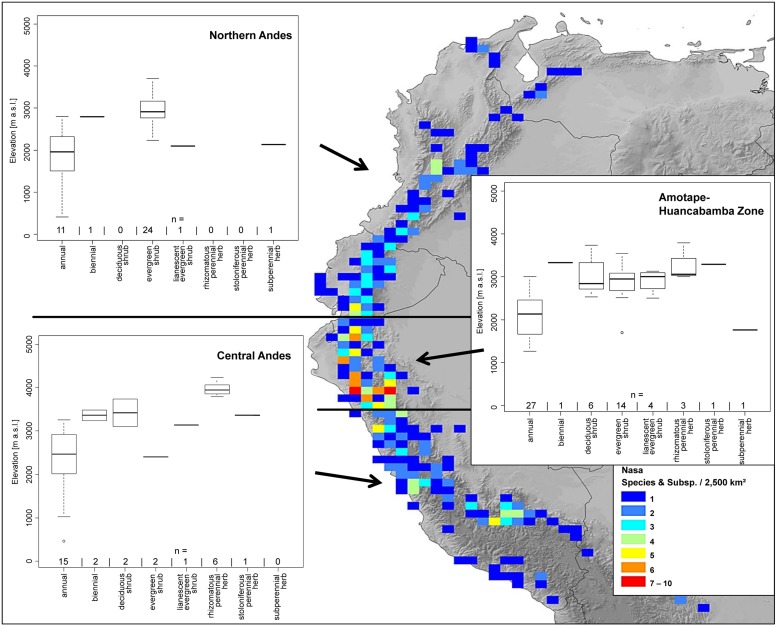
**Elevational distribution of different life forms of *Nasa* compared for the northern Andes, the Amotape–Huancabamba zone, and the central Andes.** The topography in the map is based on the GTOPO30 dataset (U.S. Geological Survey, 1996). The elevation data for the analysis is taken from the herbarium label data.

**FIGURE 6:**
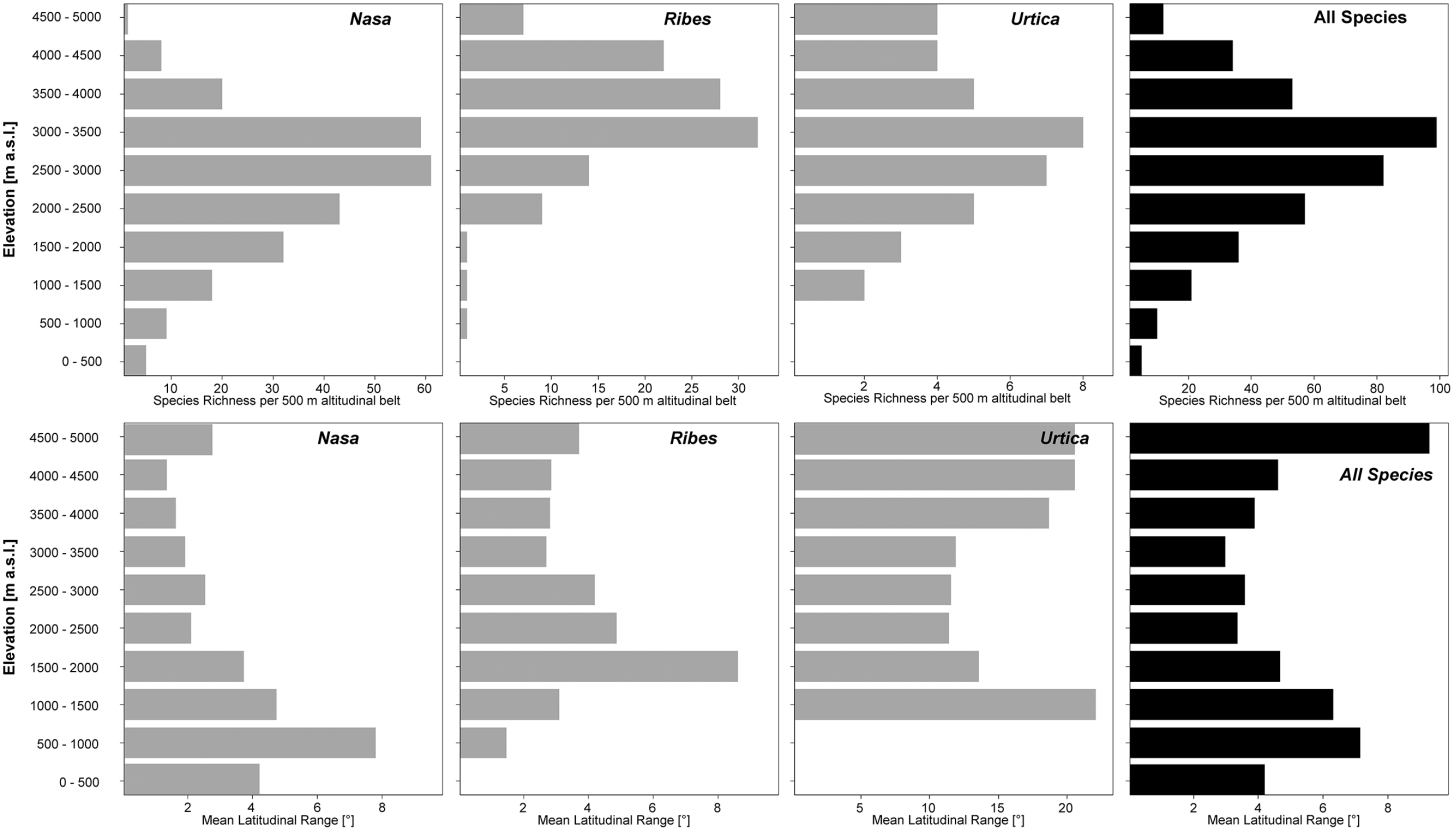
**Total species richness and mean latitudinal range per 500 m altitudinal belt of all species of *Nasa*, *Ribes* sect. *Andina*, and the “American *Urtica*” in the northern Andes, the Amotape–Huancabamba Zone, and the central Andes.** The elevation data for the analysis is taken from the original herbarium label data.

*Nasa* occurs in the tropical Andes from Venezuela to central Bolivia – only two taxa range into Central America. Most species (73 out of 98) are endemic to Ecuador and Peru. Highest species diversity in the tropical Andes is found at elevations of 2,500–3,500 m a.s.l., smallest latitudinal ranges of species are observed between 2,000 and 4,500 m a.s.l. (**Figure 3**). There is a very clear center of diversity at 6° S with over 20 taxa in a 40,000 km^2^ grid cell (**Figure 2**). Here, the most narrowly distributed taxa are found, with average latitudinal distributions of roughly 1–2°. Maximum species numbers are here found at all elevational levels between 1,000 and 4,000 m a.s.l. Species across all altitudinal bands have small latitudinal ranges. High-elevation species (mainly the *N. ranunculifolia*-group, compare **Figure 4** and Henning et al., 2011) show a slight southward displacement, with the highest species numbers found around 8–13° S, clearly paralleling the displacement to higher elevations south of the AHZ in *Ribes* (**Figure 3**). Overall, the taxa at the southern and especially the northern (13–24° N) distribution limit of the genus have comparatively wider latitudinal ranges. The highest morphological diversity with all known growth forms of the genus *Nasa* present can be found in the AHZ (**Figure 5**). In addition, species representing six of the eight growth forms have their median at more or less the same elevation around 3,000 m in the AHZ, showing a strong degree of altitudinal overlap. In the northern and central Andes only subsets of the growth forms known from *Nasa* are reported and these show moderate elevational segregation. Across the range, annual species are found at lower elevations, with a slight displacement toward higher elevations in the central Andes (**Figure 5**). Latitudinal and altitudinal distribution of the nine informal infrageneric groups in *Nasa* as a proxy of phyletic diversity is shown in **Figure 4**. There is broad overlap of the different groups between 3 and 8° S, only one group is exclusively found outside that region (*N. venezuelensis*-group in northern Colombia and Venezuela, Weigend, 1996). The highest species diversity is found at 2,500–3,500 m, and the mean latitudinal range of the species consistently is less than 3° in the altitudinal bands above 2,000 m (**Figure 6**).

*Ribes* sect. *Andina* is most diverse in the northern and central Andes (10° N and 20° S, 43 species), including the AHZ. It has several peaks of diversity along the latitudinal gradient (**Figure 3**: eight spp. per 1° latitudinal band) at 1° S, 7° S, 9° S and 13° S, respectively. Species richness in 200 km × 200 km-grid cells is highest in the AHZ in northern Peru (**Figure 2**) with up to eight species co-occurring. Between 6 and 12° S there are particularly many taxa with relatively smaller ranges (**Figure 2**), whereas the southern distribution limit is represented by a single, widespread taxon (18–23° S). Of the 43 species, 35 are restricted to elevations above 2,000 m a.s.l., half of the species reach elevations of more than 4,000 m, with the highest elevations reached between 10° S and 15° S. Highest diversity and smallest latitudinal ranges in the tropical Andes are found at elevations of 3,000–4,500 m (**Figures 3** and **6**). *Ribes* sect. *Parilla* shows a contrasting pattern, with diversity peaking at middle elevations (ca. 1,500 m) around 37° S, but species across the altitudinal and latitudinal range are generally widespread. Across its range, mean range sizes of the species are roughly one order of magnitude larger than those of sect. *Andina*—while narrow endemics play a prominent role in sect. *Andina*, they are of subordinate importance in sect. *Parilla*.

*Urtica* lacks a clear center of diversity and the majority of species across the range are widely distributed. There are two peaks of both endemicity and diversity at 7–8° S and 16–17° S (**Figures 2** and **3**). The only area with relatively narrowly distributed taxa (*U. peruviana, U. lalibertadensis, U. urentivelutina*) and a high overall species number is the AHZ around 7° S. In the southern part of the range (38–54° S) and the northern part of the South American range (6–11° N) only one widespread taxon is present (*U. magellanica* resp. *U. leptophylla*). Further north, there are five widespread species occurring mainly in Mexico. Between 16° N and 21° S, *Urtica* is virtually restricted to elevations above 2,000 m. Highest species diversity in the tropical Andes is found at elevations of 2,500–3,500 m, smallest latitudinal ranges are observed in species between 2,000 and 3,500 m (**Figure 6**). Both high and low elevation taxa are more widespread. Growth form diversity is low in *Urtica*, but all three growth forms recognized are present in the AHZ.

### IS THERE CORRESPONDENCE BETWEEN CURRENT PATTERNS OF DISTRIBUTION AND DIVERSITY WITH CLIMATE, UPLIFT HISTORY, AND TOPOGRAPHY?

**FIGURE 7:**
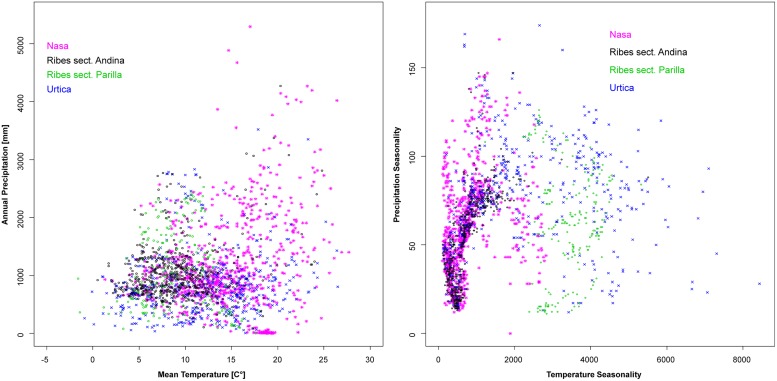
**Climatic profiles of *Nasa*, *Ribes* sect. *Andina*, *Ribes* sect. *Parilla*, and the “American *Urtica”* in Latin America based on the WORLDCLIM data set (Hijmans et al., 2005) with 30” spatial resolution.** Precipitation seasonality is defined in the dataset as the coefficient of variation of the monthly precipitation, the temperature seasonality as the standard deviation of the monthly values x100.

Climatic niches according to overall moisture and temperature plus seasonality of the two factors are shown in **Figure 7**. As mentioned in the methods section, the steep terrain of the Andes results in quality issues of the existing climate datasets which precluded more in-depth statistical analysis of these patterns. *Urtica* is the most plastic of the groups, tolerating a wide range of overall humidity and temperature conditions plus a wide range of different degrees of seasonality. The frequent co-occurrence of *Nasa*, *Urtica,* and *Ribes* sect. *Andina* in nature is reflected by their overall similar ranges in climatic preference. All three cluster at low temperature seasonality reflecting their high innertropical diversity. However, both *Urtica* and *Nasa*, to a much lesser degree *Ribes* sect. *Andina*, tolerate considerable seasonal variability in moisture. *Ribes* sect. *Parilla* is not segregated by overall temperature and moisture, but clearly segregates from sect. *Andina* in its decidedly higher adaptation to seasonal temperature variation. In comparison to *Nasa* and *Urtica*, *Ribes* sect. *Parilla* and sect. *Andina* have narrower climatic niches—separated by differences in seasonality.

The southern Andes (generally considered as the oldest part of the Andean chain) have lower diversity and especially lower endemicity in the two groups distributed along the entire Andean chain compared to the central and northern Andes (**Figures 2** and **3**). Within the tropical Andes, the AHZ (the youngest part of the Andean chain) and the central Andes immediately South of the AHZ are found to be the areas of highest species richness and narrowest endemicity in all three groups under study. In *Nasa* this is partly due to the overlap of different infrageneric groups (**Figure 4**), which in turn correlate to different life histories (and ecological niches), which accordingly are most diverse in the AHZ (**Figure 5**). A parallel, but less pronounced, situation is found in *Ribes* and *Urtica*.

**FIGURE 8:**
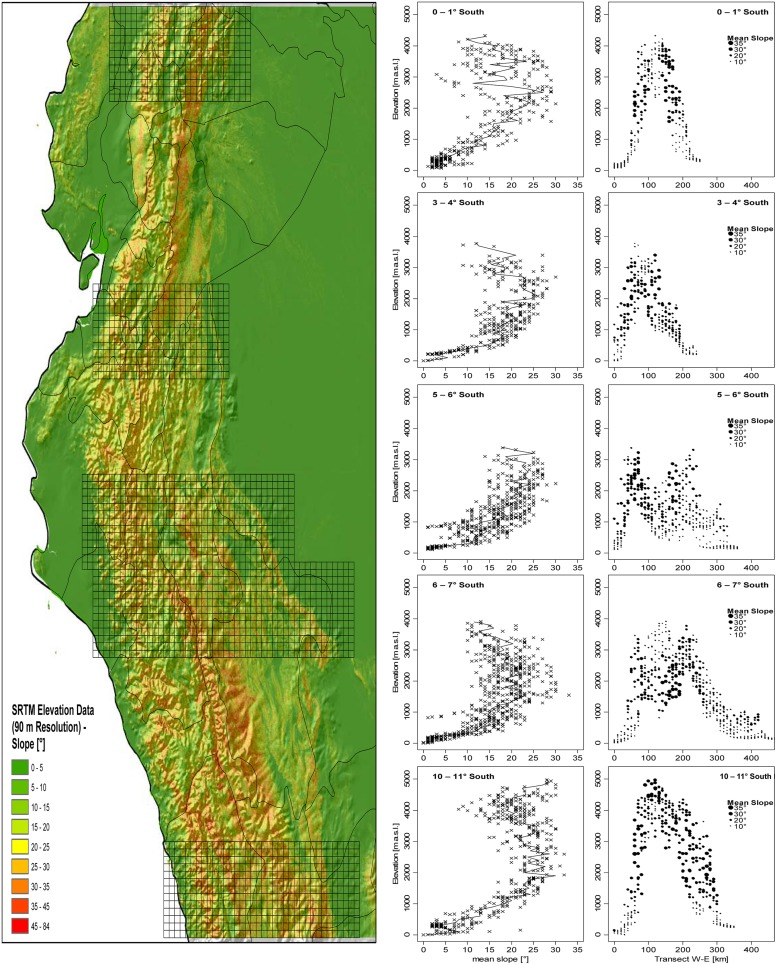
**Mean slope in relation to altitude in five East-West-Transects (each 1° North–South) in the tropical Andes.** Mean elevation and mean slope per 10 km** ×** 10 km grid cell was computed based on SRTM Elevational data with 90 m spatial resolution (Jarvis et al., 2008).

The distribution of slope inclination for five areas along the tropical Andes clearly shows that the steepest slopes are most common at elevations of 2,000–3,500 m a.s.l., roughly corresponding to the peaks of species diversity of the groups under study (**Figure 8**). Only in the Andes of central Peru (10–11° S) there is a second maximum at approximately 4,500–5,000 m.

### IS THE AMOTAPE–HUANCABAMBA ZONE A BARRIER FOR NORTH–SOUTH DISPERSAL OF MID-ELEVATION TAXA?

**FIGURE 9:**
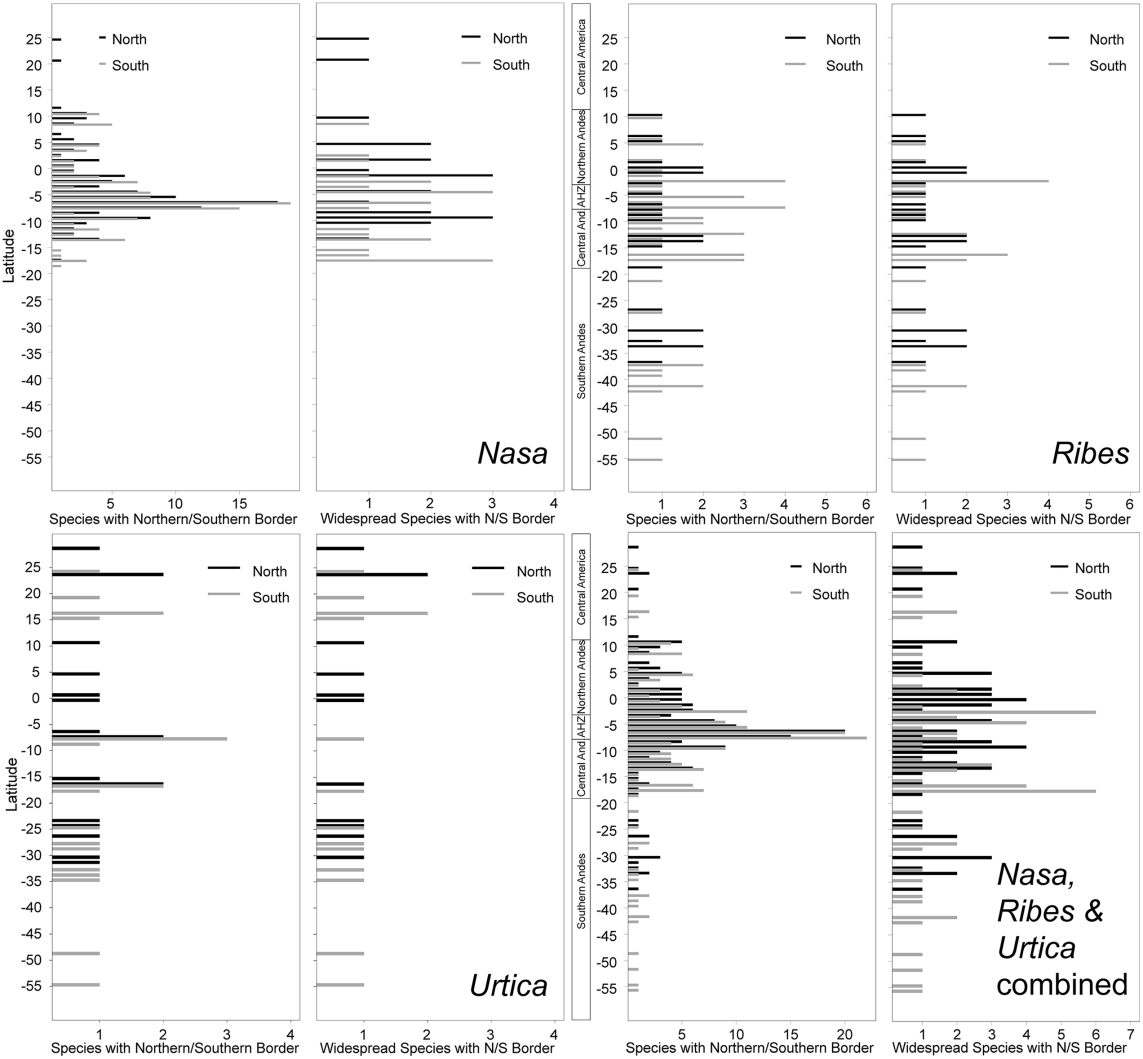
**Latitudinal positions of northern and southern range boundaries for all species of *Nasa*, *Ribes* subg. *Parilla*, and *Urtica* compared to the patters of the more widespread species with at least 2° latitudinal range (ca. 220 km)**.

The southern and northern distribution limits for the species in the groups under investigation are summarized in **Figure 9**. The graphics for overall species numbers identify no region where particularly many species reach their northern or southern limits in any of the groups. If all species are considered then there seems to be a particular “border” in the AHZ for Nasa (5–8° S), where many species reach their southern or northern limit. This is, however, entirely an artifact of the abundance of narrowly endemic taxa in this region. As soon as the analysis is restricted to the taxa with a latitudinal distribution of more than 2°, the pattern vanishes and species more or less randomly reach their limits between 5° N and 18° S. Pooling data on all 67 of these “widespread” taxa in the plant groups studied, the southern limit of the tropical Andes in Bolivia (ca. 17° S) comes out as a fairly clear southern distribution limit for six of the taxa (less than 10% of the “widespread” taxa), and there is also a southern distribution limit for the same number of taxa just north of the AHZ. However, no part of the tropical Andes is recognized as an overall important distribution limit for a major number of taxa.

## DISCUSSION

Overall latitudinal diversity patterns found here roughly show at least for *Nasa* and *Ribes* more widely distributed species toward the distribution limits of the individual groups and more narrowly distributed taxa near the equator (compare Rohde, 1992, 1999). The actual peak of diversity and restricted range taxa is displaced clearly from the equator to ca. 3–8° S. Patterns of endemicity and both altitudinal and latitudinal diversity of the three tropical Andean groups studied are remarkably similar, with *Nasa*, *Ribes,* and *Urtica* having peaks of diversity in the AHZ. The patterns for *Ribes* and *Urtica* show a displacement into higher elevations in the tropics compared to higher latitudes, as would be expected from primarily temperate plant groups. The elevational diversity patterns of the tropical Andean herb and shrub species investigated in this study show a peak at relatively high elevations of ca. 2,500–3,500 m. Latitudinal range of highest elevation taxa is not notably different from that of lower elevational bands. As shown by Braun et al. (2002) the upper forest line in the Andes reaches highest elevation at ca. 10° S of the equator, and this is roughly the region where *Ribes* and the high Andean *N. ranunculifolia*-group have their centers of diversity. This region would here technically belong to the northernmost part of the central Andes. Both the elevational band with highest species numbers and the southward displacement of maximum diversity are parallel to the patterns reported for *Puya* by Jabaily and Sytsma (2013). Previous studies, based on all angiosperms, generally found a diversity peak at lower elevations (Kessler, 2001; Braun et al., 2002; Van Der Werff and Consiglio, 2004; Jørgensen et al., 2011), which is to be expected since they all included lowland taxa.

The bulk of the herb and shrub species studied here are found at disturbed sites and represent early (annual, biennial, subperennial *Nasa*, *Urtica*) or mid- to late successional species (perennial *Urtica*, *Ribes*). Only a few of the forest species are found in climax vegetation. The peak of species diversity parallels the elevation band with the occurrence of the steepest slopes in the Andes (**Figure 8**). Different landslide risk assessments found that slope (inclination) is among the most important parameters to predict natural disturbance through landslides (e.g., Dai and Lee, 2002; Ohlmacher and Davis, 2003; Goetz et al., 2011). For the mid-elevation forests (1,900–2,800 m a.s.l.) in the Carrasco National Park, Bolivia, it is estimated that 20% of the total vascular plant flora depends on early successional vegetation types induced by landslides (Kessler, 1999). Landslides create new habitat islands, which are likely the driving force behind species survival, but also species isolation (founder effect, eco-geographical isolation) in the taxa here studied. A crucial role of cloud forest dynamics due to landslides for creating and maintaining biodiversity has been repeatedly invoked (Kessler, 1999; Nöske et al., 2008; Restrepo et al., 2009; Richter et al., 2009) and the data here presented underscore this point for the taxa under study on a regional scale. Temporal dynamics of landscape heterogeneity is thus likely an important factor in Andean diversification, with different adjacent habitat fragments representing different successional stages and housing different species complements under the same climatic and similar edaphic conditions. The AHZ of southern Ecuador and northern Peru also experiences the strongest influence of the El Niño Southern Oscillation (ENSO) resulting in subdecadal shifts in precipitation (Young, 2012), and this may add an additional aspect of temporal heterogeneity and dynamics, increasing diversity in this region.

One additional reason for differences in the levels of diversity can be deduced from the data on life history as proxy for ecological niches in the species studied: the zone with the highest species diversity also has the widest range of different growth forms, and the altitudinal differentiation of growth forms typical of other parts of the Andes is not realized (**Figure 5**): in the AHZ, typical “Puna” species (rhizomatous, perennial herbs) co-exist with typical “subpáramo” species (evergreen shrubs) in *Nasa* in the same elevational band. This reflects the fact that in this part of the Andes small-scale differences in humidity and temperature lead to a habitat mosaic, permitting the co-existence of taxa with dramatically different climatic preferences in immediately neighboring, but climatically well differentiated habitat islands, often at similar or identical elevations (Richter et al., 2009). The formation of a habitat mosaic, with individual forest fragments separated by more arid corridors is directly reflected in the presence of numerous micro-allopatric, but ecologically similar taxa, e.g., in *Nasa* (e.g., Dostert and Weigend, 1999; Henning and Weigend, 2009c). Analyzing diversity patterns of all ca. 1,400 species of Cacti, Barthlott et al. (in press) found that the Andes of northern Peru (together with the Bolivian Andes) are also the most important center of species with extremely small distribution ranges in this plant group. In addition, the AHZ together with the Andes in southern Bolivia/northern Argentina is the region with the highest diversity at the level of Tribus. A study of Andean members of the genus *Mimosa* paint a similar picture with regard to diversification in the dry valleys of northern Peru and argues for the crucial role of topographic complexity in speciation in this region (Särkinen et al., 2011). *Mimosa* and Cacti are ecologically the diametrical opposite of the mesic groups studied here and it is thus at first highly surprising that they should also have a center of endemicity in the same region. However, the explanation is obvious: arid habitats are as much isolated by corridors of mesic habitats as conversely the mesic habitats are isolated from each other by dry valleys and arid habitats. Both gross habitat types are thus present in a highly fragmented mosaic landscape pattern and provide mutually complementary conditions for micro-allopatric diversification.

It has been argued, that the geological history of the Andean chain is directly reflected in patterns of diversification and endemicity of Andean plant groups (Antonelli et al., 2009; Antonelli and Sanmartín, 2011). Under this reasoning, high Andean taxa would be expected to show elevated species numbers and high numbers of endemics in the oldest parts of the Andes, with a lower degree of diversity in younger parts of the Andean chain. In the three tropical Andean groups under study, the opposite is true: the last part of the Andean chain to rise was the AHZ and here we find the largest number of species, including endemics (and infrageneric groups in *Nasa*, indicating high phyletic diversity). Direct phylogenetic evidence for this zone being a center of phyletic diversity was also found by Struwe et al. (2009) in *Macrocarpaea* and by Smith and Baum (2006) in Iochrominae (Solanaceae), underscoring this counterintuitive finding. Recent publications, such as Cosacov et al. (2009) and Richter et al. (2009) persist in citing the actual Huancabamba Deflection (lowest pass: 5.5° S) as a dispersal barrier for Andean taxa. The analysis here presented does not find any generalized range limits either around 5.5° S or anywhere in the AHZ. Smith and Baum (2006), Cosacov et al. (2009), and Struwe et al. (2009) all show that there were several dispersal events across the Huancabamba Deflection, and the data here presented clearly show that this is a region of overlap between a range of different groups and a center of diversity, but that—based on the georeferenced data here used—a general distribution limit or dispersal barrier in this region is not retrieved. Detailed specimen based studies, especially in high Andean plant groups, should therefore re-investigate this presumed barrier critically in the future. Additionally, detailed phylogenetic investigations of a range of different plant groups, representing various habitats and elevational bands, could help to elucidate the historical processes of diversification in more detail.

## Conflict of Interest Statement

The authors declare that the research was conducted in the absence of any commercial or financial relationships that could be construed as a potential conflict of interest.
